# Correlation for the Viscosity of Methane (CH_4_) from the Triple Point to 625 K and Pressures to 1000 MPa

**DOI:** 10.1007/s10765-025-03690-7

**Published:** 2025-12-08

**Authors:** Sofia G. Sotiriadou, Konstantinos D. Antoniadis, Marc J. Assael, Viktor Martinek, Marcia L. Huber

**Affiliations:** 1 Chemical Engineering Department, Aristotle University, 54636 Thessaloníki , Greece; 2 Chemical Engineering Department, University of Western Macedonia, 50100 Kozani, Greece; 3 Interdisciplinary Center for Scientific Computing, Heidelberg University, 69120 Heidelberg, Germany; 4 Applied Chemicals and Materials Division, National Institute of Standards and Technology, 325 Broadway, Boulder, CO 80305 USA

**Keywords:** Methane, Transport properties, Viscosity

## Abstract

A wide-ranging model for the viscosity surface of methane (CH_4_) was developed with a range of validity from the triple-point temperature to 625 K and pressures up to 1000 MPa. An extensive literature survey was undertaken and all available experimental data, to the extent of our knowledge, were considered in the development of the model. The correlation incorporates recent *ab initio* results for the dilute-gas contribution, Rainwater–Friend theory for the initial density dependence, and an empirical contribution for higher densities obtained using recently developed open-source symbolic regression software. The estimated uncertainty of the correlation (at *k* = 2) varies from a low of 0.13 % for the gas at pressures below 1 MPa over temperatures from 210 K to 392 K, to 0.8 % to 2 % depending on the temperature for the mid-pressure range of 1 MPa < *p* < 50 MPa, and is 4 % for pressures from 50 MPa to 1000 MPa for temperatures from 223 K to 625 K. In the liquid region at pressures up to 33 MPa, the estimated uncertainty is 3 %.

**Supplementary Information:**

The online version contains supplementary material available at 10.1007/s10765-025-03690-7.

## Introduction

Methane is a fluid of great industrial importance since it is the main component of natural gas. In addition, it also can be used in the calibration of flow meters used in semiconductor manufacturing. Flow meters are often calibrated with one gas, and then calibration coefficients for other gases are determined using gas-property data [1], including viscosity. There are numerous [2–7] wide-ranging correlations for the viscosity of methane that cover the entire fluid range from the triple point to above the critical point, including gas, liquid, and supercritical states. Recent advances in quantum-chemical *ab initio* computations [8–10] allow improvement in the representation of the dilute-gas viscosity of methane. It is our goal to incorporate *ab initio* results to develop an improved correlation for the viscosity of methane that is valid over the entire fluid range incorporating gas, liquid, and supercritical regions.

The viscosity correlation currently incorporated in REFPROP [11] is from the unpublished work of Quiñones-Cisneros et al. [7] from 2011, and is based on generalized friction theory and critically evaluated measurements. The correlation reproduces most of the primary data close to or within the reported uncertainty:

- under 0.3 % for temperatures between 200 K and 400 K and pressures up to 30 MPa and
- up to 2 % over the fluid surface to 100 MPa for temperatures to 625 K.
- For extended pressures the uncertainty increases to 5 % for 100 MPa to 500 MPa and
- to 10 % from 500 MPa to 1000 MPa for temperatures up to 625 K.

The model extrapolates in a physically reasonable manner and may be used at pressures up to 1000 MPa and temperatures from the triple point-temperature to 1000 K. As the correlation of Quiñones-Cisneros et al. [7] was primarily based on the best available experimental data at the time (2011), it was limited by the uncertainty of those measurements. The goal of this work is to develop a new viscosity correlation that incorporates the new dilute-gas limit *ab initio* calculations for viscosity, to improve the behavior where data are unavailable and to lower the uncertainty in the dilute-gas region. In addition, we include new critically assessed literature data that became available after 2011.

In a series of papers published over the last ten years, new reference correlations over extended temperature and pressure ranges have been developed for the viscosity of some simple fluids (nitrogen [12], argon [13], xenon [14]), hydrocarbons (*n*-hexane [15], *n*-heptane [16], *n*-undecane [17], benzene [18], toluene [19], cyclopentane [20]), alcohols (ethanol [21]), glycols (ethane-1,2-diol [22], propane-1,2-diol [23]), other polar fluids (ammonia [24]), water [25] and heavy water [26]), and refrigerants (R-1234yf and R-1234ze(E) [27], R-134a [28], R-161 [29], and R32 [30]). In this paper, the methodology adopted in these papers is extended to developing a new reference correlation for the viscosity of methane.

The analysis we use begins with a critical assessment of the experimental data. We define two categories of experimental data: primary data, employed in the development of the correlation, and secondary data, used simply for comparison purposes. According to the recommendation adopted by the Subcommittee on Transport Properties (now known as The International Association for Transport Properties) of the International Union of Pure and Applied Chemistry, the primary data are identified by a well-established set of criteria [31]. These criteria have been successfully employed to establish standard reference values for the viscosity and thermal conductivity of fluids over wide ranges of conditions, with uncertainties in the range of 1 %. However, in many cases, such a narrow definition unacceptably limits the range of the data representation. Consequently, within the primary dataset, it is also necessary to include results that extend over a wide range of conditions, albeit with a poorer accuracy, provided they are consistent with other more accurate data or with theory. In all cases, the accuracy claimed for the final recommended data must reflect the estimated uncertainty in the primary information.

The form of correlation we use expresses the viscosity as a function of temperature and density. Experimental data are generally reported in terms of pressure and temperature and an equation of state (EOS) is needed to obtain corresponding densities. We use the Helmholtz EOS published by Setzmann and Wagner [32] to obtain the density for a given temperature–pressure state point. We also use the critical and triple point associated with this EOS; the critical point and other constants for this EOS are given in Table 1. The uncertainty in density of the EOS is 0.03 % for pressures below 12 MPa and temperatures below 350 K, and 0.03 % to 0.15 % for higher pressures and temperatures. The range of validity of this EOS is from the triple-point temperature to 625 K and pressures up to 1000 MPa.

**Table 1:** Critical point and fixed constants for the EOS of Setzmann and Wagner [32]

Property	Symbol	Units	Value
Critical temperature	*T*_c_	K	190.564 ± 0.012
Critical pressure	*p*_c_	MPa	4.5992 ± 0.002
Critical density	*ρ*_c_	kg·m^−3^	162.66 ± 0.2
Triple-point temperature	*T*_tp_	K	90.6941 ± 0.0025
Molar mass	*M*	g·mol^−1^	16.0428
Molar gas constant	*R*	J·mol^−1^·K^−1^	8.31451

## The Viscosity Correlation

The viscosity *η* can be expressed [14–30] as the sum of four independent contributions,

1 $$\eta \,\left( {\rho ,{\ T}} \right) = \eta_{0} {\kern 1pt} \left( {\ T} \right) + \eta_{1} {\kern 1pt} \left( {\ T} \right)\rho + {\Delta }\eta \left( {\rho ,{\ T}} \right) + {\Delta }\eta_{c} {\kern 1pt} \left( {\rho ,{\ T}} \right),$$

where *ρ* is the molar density, *T* is the absolute temperature, and the first term, *η*_0_(*Τ*) = *η*(0,*Τ*), is the contribution to the viscosity in the dilute-gas limit, where only two-body molecular interactions occur. The linear-in-density term, *η*_1_(Τ)*ρ*, known as the initial density dependence term, can be separately established using Rainwater–Friend theory [33–35] for the transport properties of moderately dense gases. The critical enhancement term, Δ*η*_c_(*ρ*,*Τ*), arises from the long-range density fluctuations that occur in a fluid near its critical point, which contribute to divergence of the viscosity at the critical point. This term for viscosity is significant only in the region very near the critical point, as shown in Vesovic et al. [36] and Hendl et al. [37] For CO_2_, Vesovic et al. [36] showed that the enhancement contributes greater than 1 % to the viscosity only in the small region bounded by 0.986 < *T*_r_ < 1.019 and 0.642 < *ρ*_r_ < 1.283 (where *T*_r_ and *ρ*_r_ denote the reduced temperature *T*_r_ = *T*/*T*_c_ and reduced density *ρ*_r_ = *ρ*/*ρ*_c_). Since data close to the critical point were unavailable, Δ*η*_c_(*ρ*,*Τ*) is be set to zero in Eq. 1 and not discussed further. Finally, the term Δ*η*(*ρ*,*T*), the residual term, represents the contribution of all other effects to the viscosity of the fluid at elevated densities including many-body collisions, molecular-velocity correlations, and collisional transfer.

The identification of these four separate contributions to the viscosity and to transport properties in general is useful because it is possible to some extent to treat *η*_0_(*Τ*), and *η*_1_(*Τ*) theoretically. In addition, it is possible to derive information about both *η*_0_(*Τ*) and *η*_1_(*Τ*) from experiment. In contrast, there is little theoretical guidance concerning the residual contribution, Δ*η*(*ρ*,*Τ*), and therefore its evaluation is based entirely on an empirical equation obtained by fitting experimental data.

In addition to performing literature searches and using content in previous correlations, we made extensive use of the NIST ThermoData Engine [38] to identify data sources. Table 2 summarizes, to the best of our knowledge, the experimental measurements of the viscosity of methane reported in the literature. Note that when processing the data, when necessary, we first convert temperatures to ITS-90 [39, 40]. In the primary dataset, with few exceptions, we included only measurements where the technique employed, and the uncertainty of the measurement are specified. Very few datasets specifically state if the uncertainty is on a *k* = 1 or *k* = 2 basis; we assume *k* = 2 when no information is given. Furthermore, with the exceptions discussed below, we preferred measurements with uncertainty less than 1 %. In the remainder of this manuscript, all uncertainties are at the *k* = 2 level unless specified otherwise.

**Table 2:** Viscosity measurements of CH_4_

Authors	Year	Method^a^	Purity (%)	Uncertainty (%)	Number of data	Temp range (K)	Press range (MPa)
*Primary data*							
Herrmann and Vogel [41, 42]^b^	2022	VbW	99.994	0.3	345	260–360	0.1–29
Humbert et al. [43]	2020	RotB	99.9995	0.2–0.5^c^	60	298–473	0.1–2
Xiao et al. [9, 10]^d^	2020	CAP	99.9995	0.134^c^	26	211–392	0, 0.1
Abramson [44]^e^	2011	RBall	99.995	10	22	294–673	570–6260
Vogel [45]	2011	OscD	99.9995	0.15–0.2	35	289–682	0.05–0.18
El Hawary[46]	2009	RotC	99.995	0.25	175	254–473	0.1–19.8
Hurly et al. [47]	2003	GAc	na	0.5	26	293	0.1–3.3
Evers et al. [48]	2002	RotB	99.9992	0.15–0.4	61	233–523	0.1–30
Assael et al. [49]	1997	VbW	99.9	1	7	313–455	0.13–0.14
van der Gulik et al. [50]	1992	VbW	99.9995	1	125	273	1–953
Diller and Frederick [51]	1989	TorQC	99.99	2	54	294–500	3–58
Hongo et al.[52]	1988	OscD	99.95	0.3	32	298–373	0.1–5.0
Diller [53]^f^	1980	TorQC	99.99	2	116	100–300	0.6–33.1
Abe et al. [54]	1978	OscD	99.9	0.3	5	298–468	0.1
Kestin et al. [55]	1977	OscD	99.99	0.2	8	298–474	0.1
Kestin et al. [56]	1977	OscD	99.99	0.1–0.2	7	296–475	0.1
Chuang [57]	1976	CAP	97.97	0.5	36	173–273	0.4–50.1
Gough et al. [58]	1976	CAP	99.8	0.5–1	10	150–320	0.1
Kestin et al. [59]	1976	OscD	99.98	0.2	3	296–477	0.1
Timrot et al. [60]	1975	OscD	99.98	0.3	9	297–675	0.03
Maitland and Smith [61]	1974	CAP	99.7	1–1.5	7	295–1022	0.1
Slyusar et al. [62]^g^	1974	FCyl	na	4	23	91–191	0.01–4.6
Haynes [63]^h^	1973	TorQC	99.97	2	20	95–190	0.01–4.5
Hellemans et al. [64]	1973	OscD	99.99	0.1–0.3	5	298–468	0.1
Dawe et al. [65]	1970	CAP	99.9	0.5	9	293–1050	0.1
Clarke and Smith [66]	1969	CAP	99.7	0.5	9	114–374	0.002–0.008
Boon et al. [67]^i^	1967	CAP	na	1	8	91–114	0.01–0.12
Giddings et al. [68]	1966	CAP	na	0.5	100	283–411	0.1–55
Carmichael et al. [69]	1965	RotC	99.9975	0.4–1	103	278–478	0.1–35.7
Barua et al. [70]	1964	CAP	99.36	0.2	39	223–423	1–17.8
Iwasaki and Takahashi [71]	1959	OscD	99.8	1	50	298–348	0.1–51.1
de Rocco and Halford [72]	1958	CAP	na	0.5	20	211–473	0.1
*Secondary data*							
Owuna et al. [73]	2024	CAP	99.995	2–8^2σ^	49	213–323	1.2–30.2
Atilhan et al. [74]	2010	ElecP	99.9995	2	77	250–450	10–70
Ling [75]	2010	ElecP	99.98	2	790	311–486	31–172
Goodwin [76]	2009	VbP	99.999	1–5	6	296	2.1–8.2
Kobayashi et al. [77]	2007	CAP	na	2	4	293–323	0.1
van der Gulik et al. [78]	1988	VbW	99.9995	0.1–0.5	40	298	0.1–1002
Knapstad [79]	1986	OscC	99.995	3–4	76	293–423	2.5–40.1
Naake [80]	1984	OscD	99.9995	1	6	291–469	11–62.3
Diaz Pena and Cheda [81]	1975	CAP	99.98	na	9	303–403	0.1
Golubev and Nazarenko [82]	1975	CAP	99.92	1.7	66	288–418	9.8–458.6
Rakshit et al. [83]	1973	OscD	99.5	1	4	238–308	0.1
Meerlender and Aziz [84]	1972	CAP	99.9	na	6	293–353	0.1
Kestin et al. [85]	1971	OscD	99.99	0.1	2	296, 302	0.1
Hellemans et al. [86]	1970	OscD	na	2	56	97–187	0.04–9.7
Kestin and Yata [87]	1968	OscD	99.99	1	16	293–303	0.1–2.6
Gonzalez et al. [88]	1967	CAP	99.8	1	53	311–444	1.4–55.2
Huang et al. [89]	1966	FCyl	99	2	90	103–273	0.1–34.5
Chakraborty and Gray [90]	1965	CAP	99.8	1	3	298–353	0.1
Martin et al. [91]	1965	CAP	na	na	8	303	14–50
Boon and Thomaes [92]	1963	CAP	99.98	1	8	91–114	0.01–0.1^f^
Senftleben [93]	1960	CAP	na	na	12	273–723	0.1
Swift et al. [94]	1960	FCyl	98.99	4	10	133–191	0.6–4.7
Baron et al. [95]	1959	CAP	99	1	40	325–408	0.7–55.2
Kestin and Leidenfrost [96]	1959	OscD	99.62	0.1	7	294–296	0.1–7.9
Swift et al. [97]	1959	FCyl	na	8	24	123–197	2.4–4.9
Pavlovich and Timrot [98]	1958	CAP	na	3	33	112–323	2–19.6
Ross and Brown [99]	1957	CAP	98.9	1	77	223–298	7–69
Lambert et al. [100]	1955	CAP	99	1	4	308–350	0.1
Uchiyama [101]	1955	CAP	na	na	4	111–473	0.1
Meshcheryakov and Golubev [102]	1954	CAP	99.8	3	170	258–523	0.1–81.1
Carr [103]	1953	CAP	99.8–99	1–2	77	294–366	0.1–55.4
Kuss [104]	1952	CAP	97	2	48	293–353	0.1–60.8
Stewart [105]	1952	CAP	99	2	39	295–340	0.8–55.4
Majumdar and Oka [106]	1949	CAP	na	na	1	273	0.1
Comings et al. [107]	1944	CAP	99.5	2	41	303–368	0.1–17.3
Bicher and Katz [108]	1943	RBall	99.3	3–8	45	298–498	0.1–34.5
Gerf and Galkov [109]	1941	OscC	na	3	5	131–181	0.4–3.4^f^
Wobser and Müller [110]	1941	RBall	na	0.5	5	293–371	0.1
Bresler and Landerman [111]	1940	CAP	na	0.5	1	90	0.1
Gerf and Galkov [112]	1940	CAP	98–99	2.5	5	94–111	0.1
Johnston and McCloskey [113]	1940	OscD	na	0.3–0.8	38	90–300	0.01–0.1
van Itterbeek [114]	1940	OscD	na	1	12	78–321	0.1
Rudenko [115]	1939	RotC	99.9	2	6	111–168	0.1–2.2
Sage and Lacey [116]^j^	1938	RBall	na	na	19	310–377	0.1–17.8
Adzumi[117]	1937	CAP	na	na	10	293–373	0.1
Rudenko and Schubnikow [118]	1934	CAP	na	na	5	90–111	0.1
Trautz and Sorg [119]	1931	CAP	na	na	6	293–523	0.1
Trautz and Zink [120]	1930	CAP	na	na	4	297–773	0.1
Ishida [121]	1923	OilD	na	0.3	1	296	0.1
Rankine and Smith [122]	1921	CAP	Pur	na	3	273–373	0.1
Vogel [123]	1914	OscD	na	4	3	96–273	0.02–0.08

^a^*CAP* capillary, *ElecP* electromagnetic piston, *FCyl* falling cyliner, *GAc* greenspan acoustic, *OilD* oil drop, *OscC* oscillating cup, *OscD* oscillating disk, *RBall* rolling ball, *RotB* rotating body, *RotC* rotating cylinder, *TorQC* torsional quartz crystal, *VbW* vibrating wire, *VbP* vibrating plate; *na* not available

^b^ Data of Schley et al. [42] were reevaluated by Herrmann and Vogel [41]

^c^Uncertainty either explicitly stated or converted to the 95 % confidence level

^d^ Data of May et al. [9] were reevaluated by Xiao et al. [10]

^e^ All but 2 points exceed the pressure and/ or temperature range of the EOS, data used to guide high pressure extrapolation behavior

^f^ Data above 180 K are secondary; 74 points included in primary

^g^ Data above 180 K are secondary; 15 points included in primary

^h^ Data above 180 K are secondary; 17 points included in primary

^i^ Saturated liquid

^j^ Data captured from graph

Hence, in the primary sets we included.

1. All measurements with stated uncertainty equal to, or less than, 1 % (or 2–2.1 % at *k* = 2).
2. We included some measurements with larger uncertainties than 1 %, or unspecified uncertainties, to extend the primary dataset to higher pressures (Diller and Frederick [51], Abramson [44]), or low temperatures (Diller [53], Slyusar et al. [62], and Haynes [63]). These were included with weights adjusted so that the fit was not overly influenced by them. Abramson [44] did not specify an uncertainty; we assigned the data an estimated uncertainty of 10 % and used the data mainly to guide extrapolations to very high (6 GPa) pressures since most of the points in the dataset exceed the range of the EOS.

The following points about data usage should also be made:

- The specific measurements of the group of Kestin performed in 1972 and earlier (Kestin et al. [85], Kestin and Yata [87], and Kestin and Leidenfrost [96]) with the instrument originally constructed by DiPippo [124, 125], as was pointed out both by Vogel [126] and Maitland et al. [127], are subject to a temperature error, and hence were not included in the primary dataset.
- The 1988 measurements of van der Gulik et al. [78] as well as the 1963 measurements of Boon and Thomaes [92] were not included in the primary dataset as they were retaken in 1992 (van der Gulik et al. [50]) and 1967 (Boon et al. [67]), respectively.
- Finally, some measurements that showed large deviations from all other sets were also not included in the primary dataset (Naake [80], Rakshit et al. [83], Gonzalez et al. [88], Chakraborty and Gray [90], Baron et al. [95], Ross and Brown [99], and Lambert et al. [100]). Measurements taken before 1941 were also not included in the primary dataset as no purity was given, or the uncertainty quoted was larger than 1 %.

All remaining measurements were considered as secondary, as they did not satisfy the aforementioned criteria.

**Fig. 1:**
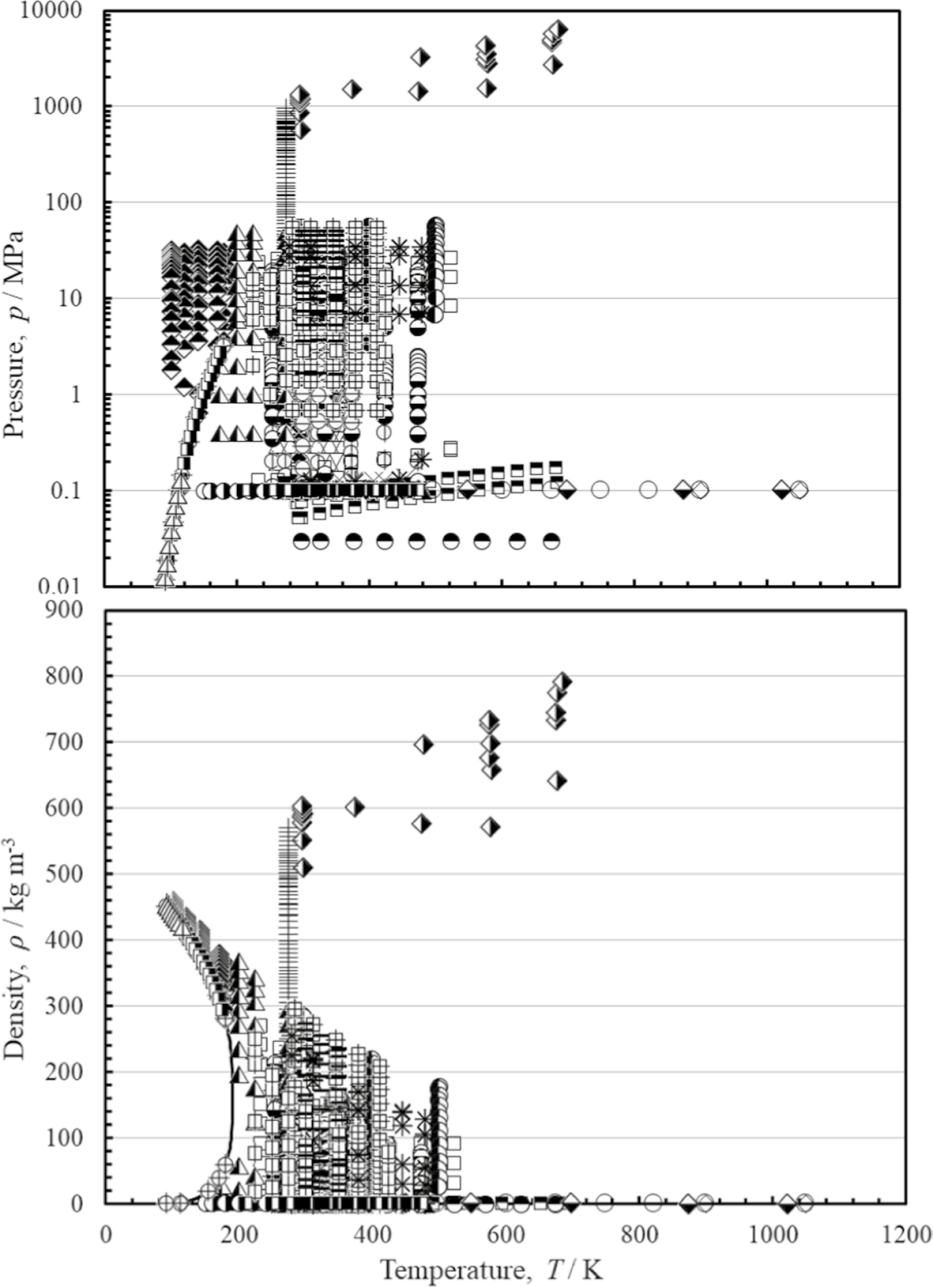
Temperature–pressure (top) and temperature-density (bottom) ranges of the primary experimental viscosity data for methane, (––) saturation curve. Herrmann and Vogel [41] (∆), Humberg et al. [43] ( ), Xiao et al. [10] (■), Abramson [44] ( ), Vogel [45] ( ), El-Hawary [46] ( ), Hurly et al.[47] (◆), Evers et al. [48] (□), Assael et al. [49] (×), van der Gulik et al. [50] (+), Diller and Frederick [51] ( ), Hongo et al. [52] ( ), Diller [53] ( ), Abe et al. [54] ( ), Kestin et al. [55] ( ), Kestin et al. [56] ( ), Chuang et al. [57] ( ), Gough et al. [58] ( ), Kestin et al. [59] ( ), Timrot et al. [60] ( ), Slyusar et al. [62] ( ), Haynes [63] ( ), Hellemans et al. [64] (●), Maitland and Smith [61] ( ), Dawe et al. [65] (○), Clarke and Smith [66] (◊), Boon et al. [67] ( ), Giddings et al. [68] ( ), Carmichael et al. [69] ( ), Barua et al. [70] (), Iwasaki et al. [71] (–), de Rocco and Halford [72] ()

Figure 1 shows the temperature–pressure (top) and temperature-density (bottom) ranges of the primary measurements outlined in Table 2, and the phase boundary.

### The Viscosity in the Dilute-Gas Limit

The dilute-gas limit viscosity, *η*_0_(*Τ*) is a function only of temperature and can be analyzed independently of all other contributions in Eq. 1. There are numerous existing formulations for the viscosity of methane in the dilute-gas limit. The most recent are those of Vogel et al. [2], Quiñones-Cisneros et al. [7], Laesecke and Muzny [128], and Xiao et al. [10].

In 2000, Vogel et al. [2] presented a correlation for *η*_0_(*Τ*) based upon their own extremely accurate measurements and the most accurate experimental data available at that time. It is in the form of a Chapman and Enskog approximate solution of the Boltzmann equation for a monatomic gas [129, 130] with an empirical temperature-dependent function for the collision integral [130]. It has a reported uncertainty of 5 % at the triple point, agrees with their experimental data that covers 260 K to 360 K to within its experimental uncertainty of 0.3 %, and rises to 2 % uncertainty at 1000 K.

In 2011, Quiñones-Cisneros et al. [7] developed a very simple correlation also based upon the most accurate experimental data available. Although unpublished, it is available in the REFPROP computer program [11]. It is primarily based upon the measurements of May et al. [9]. The dilute-gas formulation is a simple empirical polynomial expansion in terms of reduced temperature to powers of 0.25 obtained by fitting experimental data at pressures up to 1 MPa. The correlation is valid from the triple point to 1000 K. Similar to the Vogel correlation, the best experimental data for temperatures between 200 K and 400 K are represented to within 0.3 %.

In 2017, Laesecke and Muzny [128] presented a wide-ranging formulation for *η*_0_(*Τ*) based on the *ab initio* work of Hellmann et al. [131] adjusted to agree with the experimental data of May et al. [9] that were re-analyzed with helium reference values from Cencek et al. [132]. One benefit of incorporating of *ab initio* calculations is to obtain better representation in regions where experimental data are of low quality or entirely lacking. In their work they used symbolic regression to obtain a functional form that extrapolates both to low temperature (*T* → 0) and high temperature (*T* = 10,000 K) limits. Careful attention was given to the extrapolation behavior. In their manuscript, they give detailed comparisons with experimental data and with the correlations of Vogel et al. [2] and Quiñones Cisneros et al. [7]. They demonstrate that their formulation is superior to both Vogel et al. [2] and Quiñones Cisneros et al. [7], especially at lower temperatures (below ~ 200 K) and higher temperatures (~ 800 K) in part due to their use of the *ab initio* values and their careful consideration of extrapolation behavior. They claim the estimated expanded uncertainty (*k* = 2) of the correlation is 1 % from 80 to 150 K, 0.3 % from 150 to 210 K, 0.053 % for 210 K to 392 K (the range of the May et al. [9] data), 0.2 % for 392 K to 700 K, 0.5 % for 700 K to 1100 K, and 1 % to 1500 K (the upper limit of the *ab initio* calculations).

Finally, in 2020 Xiao et al. [10] developed an expression for *η*_0_(*Τ*) also using the *ab initio* work of Hellman et al. [131]. The *ab initio* values at discrete temperatures are first divided by an *ab initio* value at a reference temperature, in this case 298.15 K, to create a ratio of the viscosity calculated *ab initio* at a temperature *T* divided by its value at a reference temperature. This ratio was then fit to a fixed polynomial form to at least one order of magnitude smaller than the uncertainties of the values in the original publication, so that the same precision as the original publication is retained. The final correlation is then obtained by multiplying by a reference value for methane at 298.15 K obtained from experiment. Xiao et al. [10] used the value recommended by Berg and Moldover [133] of 11.0631 µPa s at 298.15 K that was based on calculating viscosity ratios using critically reviewed experimental data from several experimental sources and fitting them to obtain the most accurate values at 298.15 K, and then anchoring them to the He value at 298.15 K from Cencek et al. [132]. This methodology takes advantage of the fact that viscosity ratios are more accurate than the viscosities themselves as discussed by Berg and Moldover [133]. The Laesecke and Muzny correlation [128] has a slightly different value at 298.15 K, 11.0592 µPa s obtained by recalculating May et al. [9] with the helium reference value of Cencek et al. [132].

**Fig. 2:**
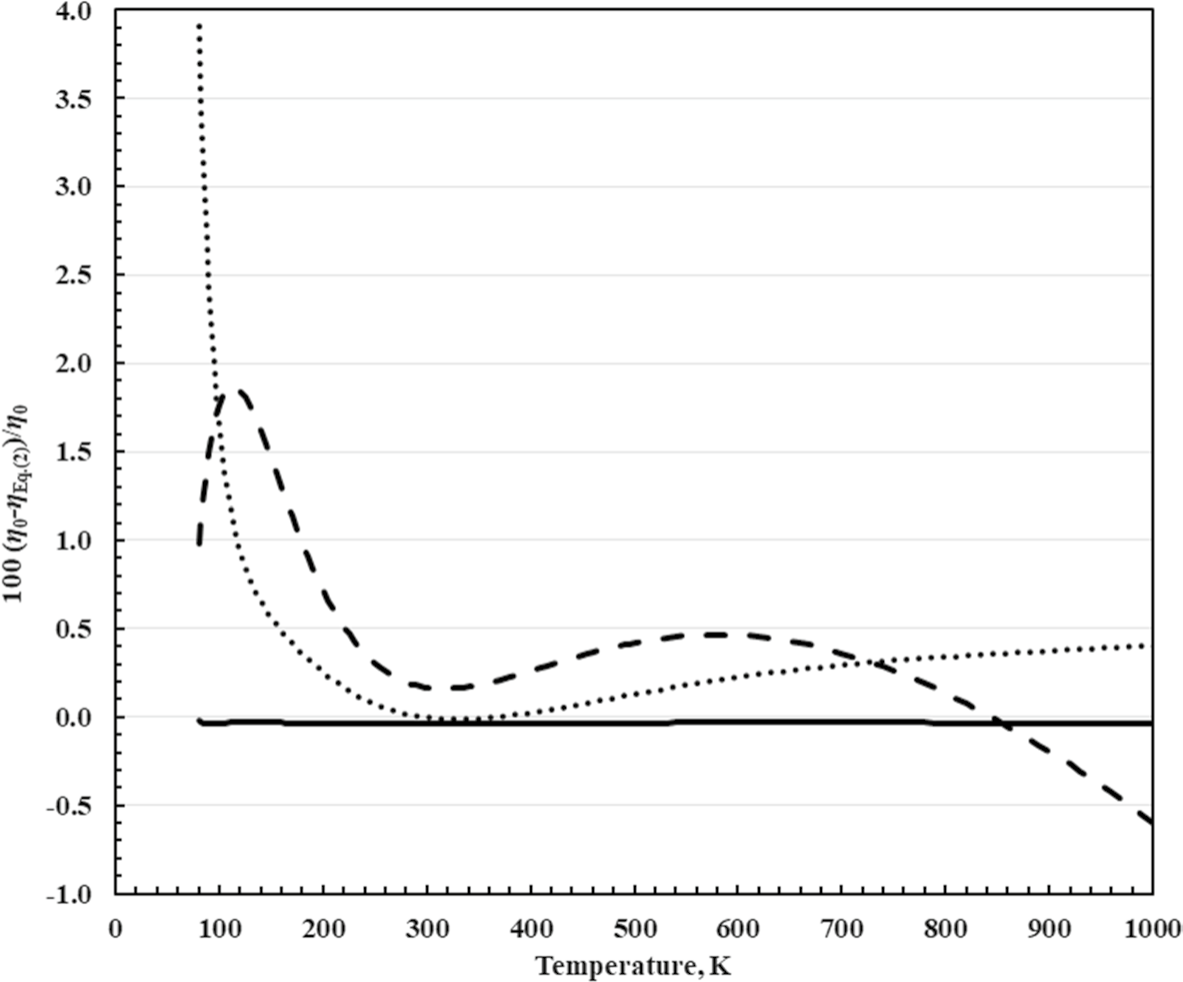
Relative deviations of the dilute-gas viscosity values *η*_0_ from Eq. 2. Quiñones-Cisneros et al. [7], dotted line; Vogel et al. [2], dashed line; Laesecke and Muzny [128], solid line

The correlation of Laesecke and Muzny [128] is recommended if one is interested in the extrapolation behavior to 0 K and the very high temperature extrapolation behavior to 10,000 K. In this work, we are interested in the practical temperature range of the triple point to 1000 K. In this region, the Laesecke and Muzny correlation [128] and the Xiao et al. correlation [10] are essentially equivalent in performance. The Xiao et al. [10] correlation has slightly better agreement with the *ab initio* values due to its replication of the precision of the *ab initio* values but the differences are insignificant for the purposes of this work. In this work we selected the dilute-gas viscosity correlation proposed by Xiao et al. [10], primarily for consistency with our recent work on nitrogen [12] and argon [13]. The Xiao et al. [10] correlation is

2 $$\eta_{0} (T) = \eta_{0} (298.15\;{\text{K}})\exp \left( {\sum\limits_{i = 1}^{8} {a_{i} } \left[ {\ln (T/298.15\;{\text{K}})} \right]^{i} } \right),$$

where *η*_0_(298.15 K) = 11.0631 μPa·s, and the coefficients *a*_*i*_ are shown in Table 3. Deviations from the correlations of Quiñones-Cisneros et al. [7], Vogel et al. [2], and Laesecke and Muzny [128] from the correlation of Xiao et al. [10] given in Eq. 2 are shown in Fig. 2. As indicated in Fig. 2, the correlations of Laesecke and Muzny [128] and Xiao et al. [10] are equivalent. The deviations for the two models that did not incorporate the most recent *ab initio* results (Quiñones-Cisneros et al. [7], Vogel et al. [2]) are largest in the very high and very low temperature regions where reliable experimental data were not available for regression.

**Table 3:** Coefficients *a*_*i*_ (-) of Eq. 2

*i*	*a*_*i*_	*i*	*a*_*i*_
1	8.73963 × 10^−1^	5	− 9.11659 × 10^−3^
2	− 1.17213 × 10^−1^	6	− 1.65050 × 10^−3^
3	3.29256 × 10^−4^	7	1.788860 × 10^−3^
4	2.65091 × 10^−2^	8	− 3.38860 × 10^−4^

### The Initial-Density Dependence Viscosity Term

The temperature dependence of the linear-in-density coefficient of the viscosity *η*_1_(*T*) in Eq. 1 is large at subcritical temperatures and must be considered to obtain an accurate representation of the behavior of the viscosity in the vapor phase. It changes sign from positive to negative as the temperature decreases. Therefore, the viscosity along an isotherm should first decrease in the vapor phase and subsequently increase with increasing density [130]. Vogel et al. [134] have shown that fluids exhibit the same general behavior of the initial density dependence of viscosity, which can also be expressed by means of the second viscosity virial coefficient *B*_η_(*T*), as

3 $$\eta_{1} ({\ T}) = \eta_{0} ({\ T}){\ B}_{\eta } ({\ T}).$$

**Fig. 3:**
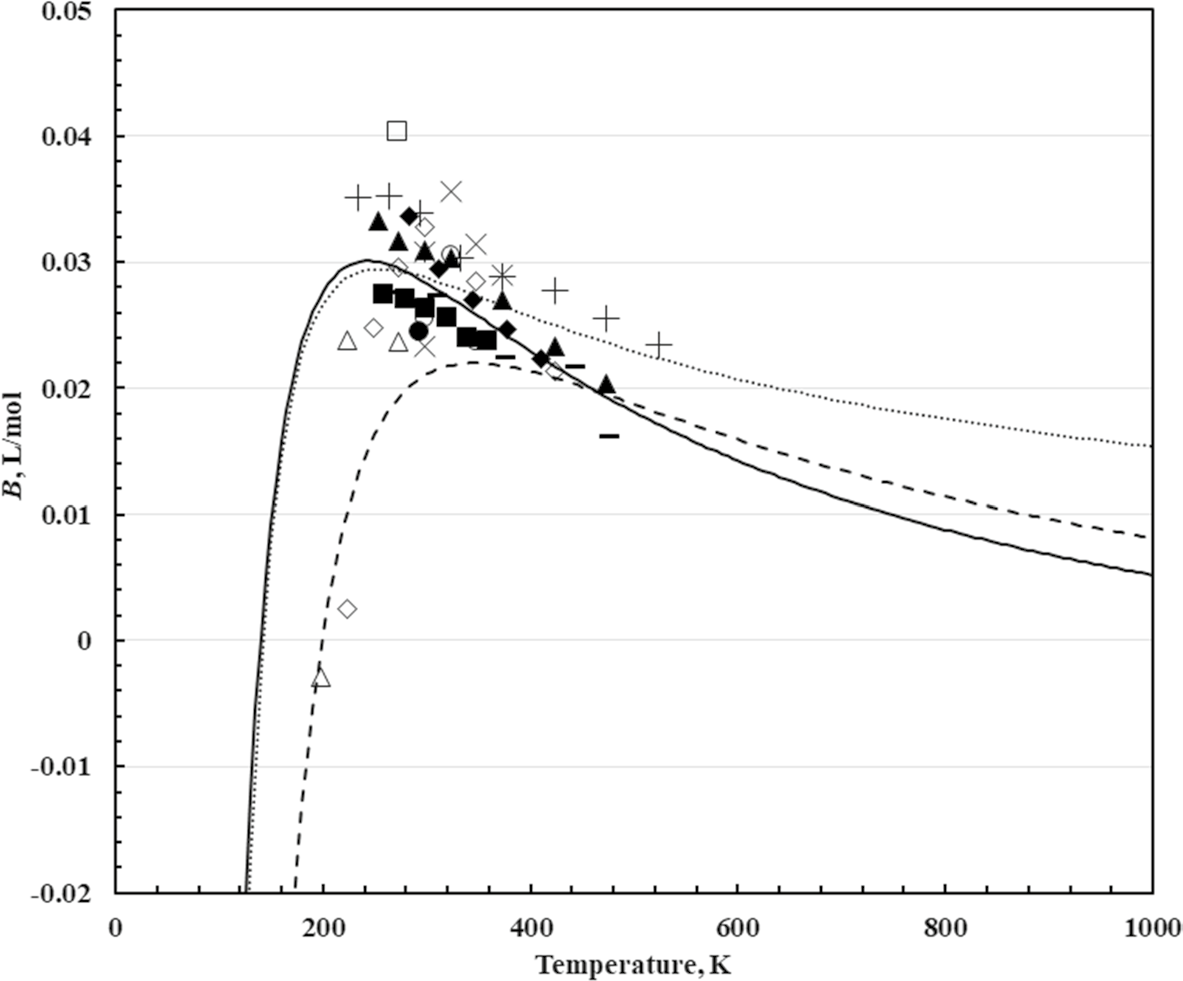
The second viscosity virial coefficient of methane, *B*, as a function of temperature. Equations 4 and 5 with LJ parameters from Vogel et al. [2], dashed line, Eqs. 4 and 5 with new LJ parameters from this work, solid line, Correlation of Quiñones-Cisneros et al. [7] dotted line, Herrmann and Vogel [41] (■), Humberg et al. [43] (▲), Berg and Moldover [133] (✳), Hurly et al. [47] (●), Evers et al. [48] (+), van der Gulik et al. [50] (□), Hongo et al. [52] (×), Chuang et al. [57] (∆), Giddings et al. [68] (◆), Barua et al. [70] (◊), Iwasaki et al. [71] (◯), Carmichael et al. [69] (**–**)

The second viscosity virial coefficient can be obtained according to the theory of Rainwater and Friend [33, 34] as a function of a reduced second viscosity virial coefficient, ${\ B}_{\eta }^{*} ({\ T}^{*} )$, as

4 $${\ B}_{\eta } ({\ T}) = {\ B}_{\eta }^{*} ({\ T}^{*} )N_{{\text{A}}} \sigma^{3} ,$$

where *N*_A_ is the Avogadro constant, *σ* is an intermolecular potential distance parameter, and T* is a scaled temperature that depends on the intermolecular potential. In this work we use the Lennard–Jones potential and an expression developed by Vogel et al. [130] for ${\ B}_{\eta }^{*} ({\ T}^{*} )$

5 $${\ B}_{\eta }^{*} ({\ T}^{*} ) = \sum\limits_{\iota = 0}^{6} {b_{i} \left( {T^{*} } \right)^{ - 0.25i} } + b_{7} \left( {T^{*} } \right)^{ - 2.5} + b_{8} \left( {T^{*} } \right)^{ - 5.5}$$

where T* is the scaled temperature *T*/(*ε*/*k*_B_). The coefficients *b*_*i*_, from Ref. [130], are given in Table 4. Vogel and coworkers [2, 35] used the Lennard Jones parameters *σ* = 0.3706 nm and *ε*/*k*_B_ = 159.7 K. Figure 3 shows experimentally derived values of *B*_*η*_ along with values computed using Eq. (5) and the Lennard–Jones parameters used by Vogel and coworkers [2, 35]. If values of *B*_*η*_ were not supplied in the original paper, we obtained them by plotting the experimental viscosity along an isotherm as a function of density, adjusting to the nominal temperature if necessary, using the temperature dependence of the dilute-gas formulation of Xiao et al. [10]. The dashed curve in Fig. 3 obtained from Eq. 5 with the Lennard–Jones parameters of Vogel and coworkers [2, 35] is consistently lower than the experimental values and the peak appears to be offset slightly from the data. We refit the Lennard–Jones parameters to be more consistent with the data and obtained the values *σ* = 0.4115 nm and *ε*/*k*_B_ = 112.2 K, shown as a solid line in Fig. 3. Also shown as a dotted line are values calculated from the correlation of Quiñones-Cisneros et al. [7]

**Table 4:** Coefficients *b*_*i*_ [130] and Lennard–Jones parameters (this work) for Eq. 5

*i*	*b*_*i*_
0	− 1.9572881 × 10^1^
1	2.1973999 × 10^2^
2	− 1.0153226 × 10^3^
3	2.47101251 × 10^3^
4	− 3.3751717 × 10^3^
5	2.4916597 × 10^3^
6	− 7.8726086 × 10^2^
7	1.4085455 × 10^1^
8	− 3.4664158 × 10^–1^
*σ* = 0.4115 nm	*ε*/*k*_B_ = 112.2 K

### The Residual Term

As stated in Sect. 2, the residual viscosity term, Δ*η*(*ρ*,*T*), represents the contribution of all other effects to the viscosity of the fluid at elevated densities including many-body collisions, molecular-velocity correlations, and collisional transfer. Since we have little theoretical guidance on the contributions at high density, we use a completely empirical approach that employs a symbolic regression technique [135, 136] to determine Δ*η*(*ρ*,*T*).

The procedure adopted during this analysis used recently developed, open-source symbolic regression software TiSR of Martinek and coworkers [135, 136] to fit the primary data to obtain the residual viscosity correlation Δ*η*(*ρ*,*T*). The functional form is not known at the start of the regression process; symbolic regression is used to determine not only the coefficients but also the functional form of the correlation. Symbolic regression is a type of genetic programming that allows the exploration of arbitrary functional forms to regress data. The functional form is obtained by use of a set of operators, parameters, and variables as building blocks. In the present work we restricted the operators to the set (+ ,–, *, /, ^, sqrt, pow2) and the operands (constant, *T*_r_, *ρ*_r_), with *T*_r_ = *T/T*_c_ and *ρ*_r_ = *ρ*/*ρ*_c_. In addition, we incoporated a form suggested from the hard-sphere model employed by Assael et al. [137] Δ*η*(*ρ*_r_,*T*_r_) = (*ρ*_r_^2/3^*T*_r_^1/2^)*F*(*ρ*_r_,*T*_r_), where the symbolic regression method was used to determine the functional form for *F*(*ρ*_r_,*T*_r_). For this task, the dilute-gas limit and the initial density dependence terms were calculated for each experimental point (employing Eqs. 2–5) and subtracted from the experimental viscosity to obtain the residual term. We started with a weight factor equal to the inverse of the uncertainty squared, but then adjusted the weights on the data as necessary to ensure the residual contribution was near zero for densities less than 2 mol·L^−1^ to retain the theoretical values. During the regression process, many possible solutions are given; as the complexity increases the value of the objective function decreases. Our goal was to obtain a solution with a minimum number of coefficients and complexity but with adequate representation of the data. We found that using a complexity factor of 30 was optimal. Additional information on the options controlling the symbolic regression are in Martinek et al. [135, 136]. The final equation obtained was

6 $${\Delta }\eta (\rho ,{\rm T}) = \left( {\rho_{{\text{r}}}^{{2/3}} T_{{\text{r}}}^{{1/2}} } \right)\left\{ {\left( {\frac{{f_{1} \rho_{{\text{r}}}^{2} + f_{2} \rho_{{\text{r}}}^{3} }}{{f_{3} + T_{{\text{r}}} - \rho_{r}^{{}} }} + \left( {f_{4} + T_{{\text{r}}}^{{f_{5} }} } \right)\left( {f_{6} \rho_{{\text{r}}}^{{}} + f_{7} \rho_{{\text{r}}}^{{2}} + f_{8} \rho_{{\text{r}}}^{3} + f_{9} \rho_{{\text{r}}}^{4} } \right)} \right)} \right\}.$$

**Fig. 4:**
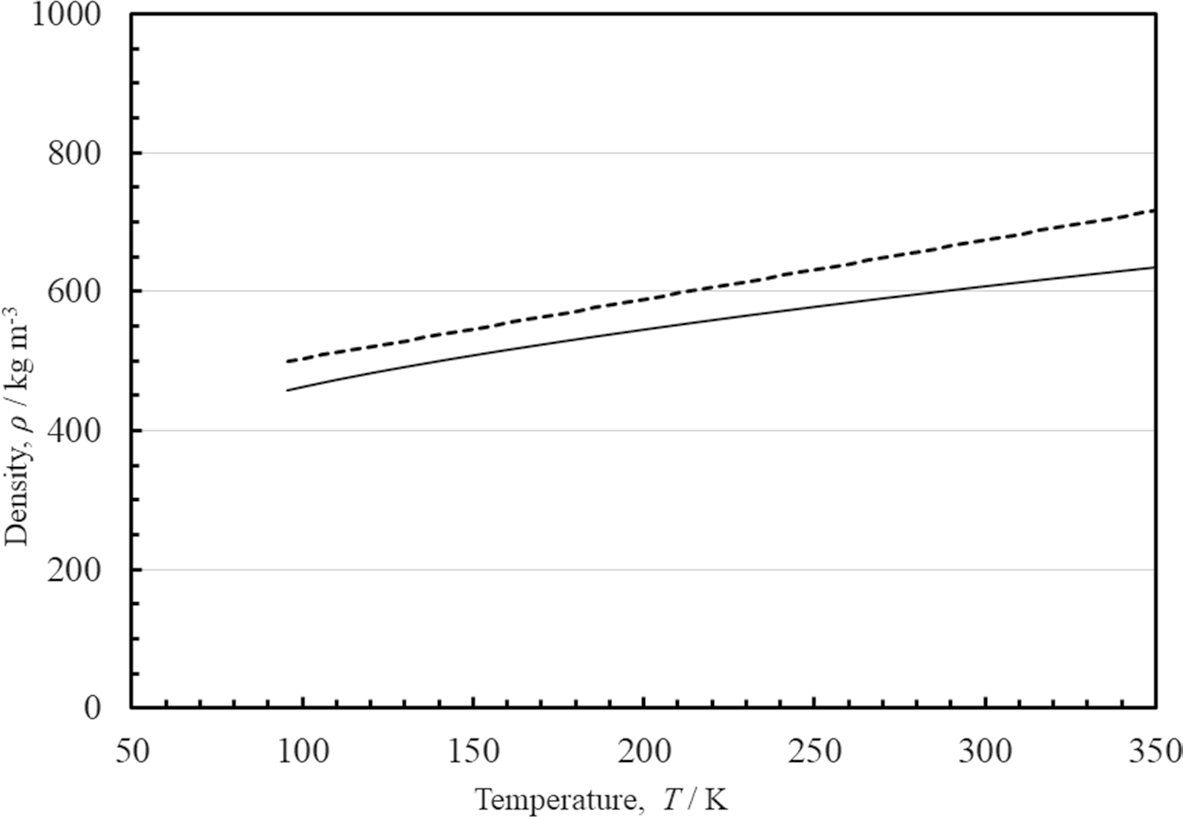
The melting line for methane and location of poles of Eq. 6. Melting line from Setzmann and Wagner [32] solid line, line of poles from Eq. 6 dashed line

The coefficients are given in Table 5, and Δ*η* is in μPa·s. A parameter file suitable for use with the NIST REFPROP [11] program is included in the supplemental information that gives the full correlation Eqs. 1–6. When using symbolic regression programs, we have noticed that the resulting correlation often has mathematical poles. This is true for the correlation here as well, as there are discontinuities when the denominator of Eq. 6 is zero. For integration into software that may encounter evaluation beyond physically meaningful conditions, we recommend users check that the region *ρ*_r_ _=_ *f*_3_ + *T*_r_ is avoided to ensure discontinuities are not encountered and cause numerical instabilities. For methane, we know the location of the melting line, and the line of poles in Eq. 6 is well into the solid region as shown in Fig. 4. The melting line is that given by Setzmann and Wagner [32]. It was developed using experimental data that covered the temperature range from the triple-point temperature to 367 K.

**Table 5:** Coefficients *f*_*i*_ for Eq. 6

*i*	*f*_*i*_
1	0.293 514
2	0.923
3	2.57
4	0.513
5	− 1.48
6	1.653 650 300 856
7	4.218 535 015 2
8	− 2.846 307 84
9	0.775 32

### Comparison with Data

The final correlation model consists of Eqs. 1–6 with the critical enhancement term set to zero. Table 6 summarizes comparisons of the primary data with the present correlation, while Table 7 gives comparisons of the secondary data. Comparisons with the correlation of Quiñones-Cisneros et al. [7] are also given. We use the following expressions for the percent deviation (PCT), average absolute relative deviation (AARD) and BIAS

7 $${\text{PCT}}_{i} = \frac{{100\Delta \eta_{i} }}{{\eta_{i} }} = \frac{{100(\eta_{\exp ,i} - \eta_{{\text{calc,i}}} )}}{{\eta_{{{\text{calc}},i}} }},$$

8 $${\text{AARD = }}\left( {\sum\limits_{i = 1}^{n} {\left| {{\text{PCT}}_{i} } \right|} } \right)/n,$$

9 $${\text{BIAS = }}\left( {\sum\limits_{i = 1}^{n} {{\text{PCT}}_{i} } } \right)/n,$$

where *n* is the number of data points, *η*_exp_ is the experimental value of the viscosity, and *η*_calc_ is the value calculated from the correlation. The maximum deviation (positive or negative) is also given.

**Table 6:** Evaluation of the methane viscosity correlation for the primary data

Investigators/reference	Year Publ	AARD (%)	BIAS (%)	MAX (%)	AARD (%)	BIAS (%)	MAX (%)
		Present work			Quiñones-Cisneros et al. [7]		
Herrmann and Vogel [41]	2022	0.11	− 0.03	− 0.29	0.16	− 0.16	− 0.54
Humbert et al.[43]	2020	0.11	− 0.10	− 0.25	0.15	− 0.14	− 0.48
Xiao et al. [10]	2020	0.04	0.01	0.11	0.03	− 0.03	− 0.09
Abramson [44]	2011	3.09	− 2.57	− 6.83	29.81	− 29.53	− 54.39
Vogel [45]	2011	0.19	0.19	0.34	0.10	0.08	0.33
El Harwary [46]	2009	0.29	0.28	1.14	0.17	0.11	0.84
Hurly et al. [47]	2003	0.18	− 0.17	− 0.28	0.17	− 0.16	− 0.28
Evers et al. [48]	2002	1.30	1.30	2.39	0.96	0.94	2.18
Assael et al. [49]	1997	0.65	0.65	0.94	0.63	0.63	0.95
Van der Gulik et al. [50]	1992	0.99	0.89	3.50	2.94	2.91	7.19
Diller and Frederick [51]	1989	2.23	1.99	7.29	1.98	1.55	7.20
Hongo et al. [52]	1988	0.76	0.76	1.16	0.71	0.71	1.16
Diller [53]	1980	1.00	0.08	2.45	1.14	− 0.51	3.92
Abe et al. [54]	1978	0.53	0.53	0.79	0.50	0.50	0.80
Kestin et al. [55]	1977	0.55	0.55	0.63	0.51	0.51	0.55
Kestin et al. [56]	1977	0.58	0.58	0.69	0.53	0.53	0.67
Chuang [57]	1976	0.81	− 0.61	− 2.58	1.08	− 1.03	− 2.83
Gough et al. [58]	1976	0.47	0.47	0.72	0.28	0.28	0.38
Kestin et al. [59]	1976	0.53	0.53	0.75	0.49	0.49	0.63
Timrot et al. [60]	1975	0.64	0.64	0.96	0.52	0.52	0.96
Slyusar et al. [62]	1974	2.97	1.38	7.66	3.26	2.51	11.46
Haynes [63]	1973	0.76	− 0.18	− 2.03	1.28	0.25	4.24
Hellemans et al. [64]	1973	0.51	0.51	0.91	0.48	0.48	0.90
Maitland and Smith [61]	1974	0.66	0.26	− 1.24	0.68	0.01	− 1.66
Dawe et al. [65]	1970	0.42	0.25	0.94	0.54	0.01	− 0.97
Clarke and Smith [66]	1969	0.89	0.89	2.18	0.64	0.64	1.38
Boon et al. [67]	1967	1.75	− 1.39	− 2.74	1.55	0.84	4.12
Giddings et al. [68]	1966	1.31	1.31	2.36	1.09	1.06	2.12
Carmichael et al. [69]	1965	1.85	1.85	4.37	1.54	1.54	4.20
Barua et al. [70]	1964	0.76	− 0.26	− 3.31	0.84	− 0.57	− 3.77
Iwasaki and Takahashi [71]	1959	0.76	0.54	2.28	0.81	0.27	2.23
De Rocco and Halford [72]	1958	1.03	1.03	2.13	0.99	0.99	2.06

**Table 7:** Evaluation of the methane viscosity correlation for the secondary data

Investigators/reference	Year Publ	AARD (%)	BIAS (%)	MAX (%)	AARD (%)	BIAS (%)	MAX (%)
		Present work			Quiñones-Cisneros et al. [7]		
Owuna et al. [73]	2024	0.83	0.10	2.00	0.58	− 0.25	− 1.76
Atilhan et al. [74]	2010	1.26	0.72	− 3.92	1.22	0.29	− 3.93
Ling [75]	2010	1.02	− 0.05	3.62	1.17	− 0.11	3.90
Goodwin [76]	2009	2.06	− 2.06	− 3.94	2.15	− 2.15	− 4.15
Kobayashi et al. [77]	2007	0.85	0.85	1.24	0.86	0.86	1.25
van der Gulik et al. [78]	1988	5.00	4.90	8.14	5.08	4.41	10.97
Knapstad [79]	1986	1.45	1.24	− 2.72	1.26	1.01	− 3.13
Naake [80]	1984	3.31	− 0.40	8.58	3.59	− 0.68	8.72
Diaz Pena and Cheda [81]	1975	0.46	− 0.03	− 0.78	0.46	− 0.03	− 0.77
Golubev and Nazarenko [82]	1975	3.60	− 0.96	29.51	3.31	− 0.44	29.21
Rakshit et al. [83]	1973	10.42	− 10.42	− 11.58	10.45	− 10.45	− 11.64
Meerlender and Aziz [84]	1972	0.34	− 0.34	− 0.61	0.34	− 0.34	− 0.60
Kestin et al. [85]	1971	0.09	0.09	0.10	0.09	0.09	0.11
Hellemans et al. [86]	1970	5.41	− 3.07	− 30.32	5.71	− 3.31	− 30.77
Kestin and Yata [87]	1968	0.19	0.19	0.33	0.20	0.20	0.33
Gonzalez et al. [88]	1967	3.08	3.08	5.75	2.82	2.82	5.71
Huang et al. [89]	1966	1.94	1.37	13.00	1.72	0.69	12.26
Chakraborty and Gray [90]	1965	0.97	− 0.97	− 2.07	0.97	− 0.97	− 2.07
Martin et al. [91]	1965	18.35	18.35	32.21	17.70	17.70	32.08
Boon and Thomaes [92]	1963	1.70	− 0.90	− 8.69	2.28	2.28	5.98
Senftleben [93]	1960	3.57	− 2.57	− 6.97	3.68	− 2.68	− 7.27
Swift et al. [94]	1960	5.55	2.99	13.00	4.84	2.16	13.58
Baron et al. [95]	1959	2.80	2.72	4.58	2.60	2.50	4.41
Kestin and Leidenfrost [96]	1959	0.72	0.68	1.07	0.71	0.61	1.08
Swift et al. [97]	1959	15.74	7.71	53.95	15.74	6.80	54.97
Pavlovich and Timrot [98]	1958	13.15	13.00	49.03	12.79	12.63	46.41
Ross and Brown [99]	1957	4.60	4.49	13.57	4.08	3.87	13.51
Lambert et al. [100]	1955	2.03	− 2.03	− 2.60	2.03	− 2.03	− 2.60
Uchiyama [101]	1955	4.37	− 4.37	− 9.15	4.60	− 4.60	− 9.25
Meshcheryakov and Golubev [102]	1954	1.11	− 0.62	− 4.07	1.35	− 0.99	− 5.21
Carr [103]	1953	1.13	0.58	2.64	1.20	0.34	− 2.62
Kuss [104]	1952	3.31	− 3.27	− 9.83	3.57	− 3.56	− 10.41
Stewart [105]	1952	2.25	2.25	6.40	1.94	1.94	5.41
Majumdar and Oka [106]	1949	4.97	− 4.97	na	4.99	− 4.99	na
Comings et al. [107]	1944	1.28	0.95	2.64	1.12	0.78	2.40
Bicher and Katz [108]	1943	5.83	5.63	17.05	5.51	5.28	16.90
Gerf and Galkov [109]	1941	32.16	32.16	71.65	31.74	31.74	68.67
Wobser and Müller [110]	1941	0.11	0.07	0.16	0.12	0.08	0.16
Bresler and Landerman [111]	1940	0.33	− 0.33	na	6.39	6.39	na
Gerf and Galkov [112]	1940	1.00	0.69	2.86	2.58	2.58	7.41
Johnston and McCloskey [113]	1940	1.46	1.46	4.71	1.09	1.09	2.83
van Itterbeek [114]	1940	4.18	4.02	13.38	2.85	2.69	8.54
Rudenko [115]	1939	28.73	23.10	57.59	28.76	23.14	57.66
Sage and Lacey [116]	1938	7.18	7.15	15.83	7.00	6.97	15.56
Adzumi[117]	1937	3.10	3.10	4.21	3.11	3.11	4.22
Rudenko and Schubnikow [118]	1934	7.00	− 6.82	− 16.92	9.63	− 3.61	− 16.88
Trautz and Sorg [119]	1931	0.74	− 0.74	− 1.27	0.79	− 0.79	− 1.43
Trautz and Zink [120]	1930	1.27	− 1.27	− 2.43	1.47	− 1.47	− 2.77
Ishida [121]	1923	2.01	− 2.01	na	2.01	− 2.01	na
Rankine and Smith [122]	1921	1.30	1.30	1.78	1.28	1.28	1.77
Vogel [123]	1914	3.09	− 0.85	− 5.92	3.54	− 1.50	− 7.57

**Fig. 5:**
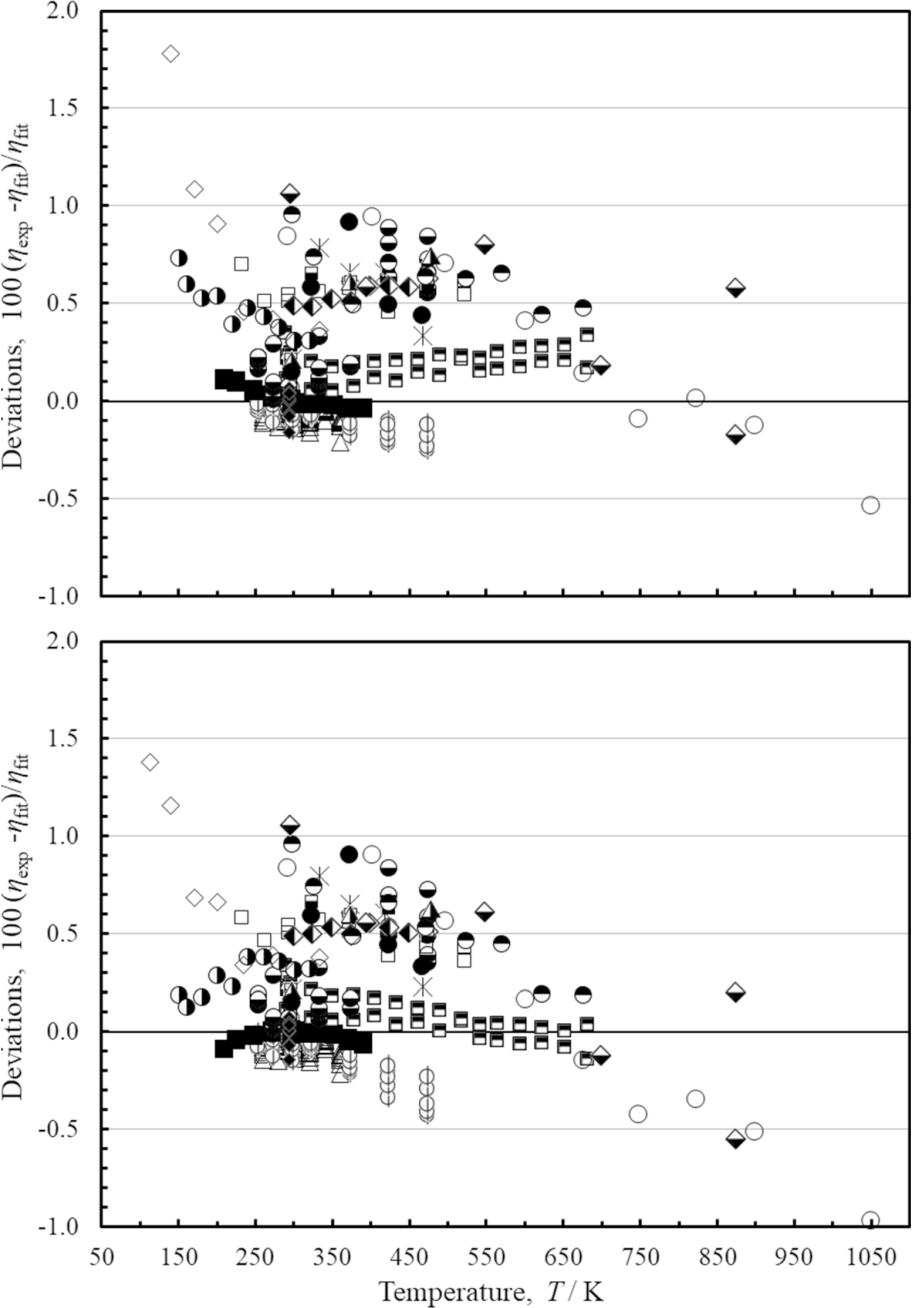
Percentage deviations of selected data at low pressures (*p* ≤ 1 MPa) calculated by the present model and the model of Quiñones-Cisneros et al. [7]. Top panel, this work, bottom panel, Quiñones-Cisneros et al. [7]. Herrmann and Vogel [41] (∆), Humberg et al. [43] ( ), Xiao et al. [10] (■), Vogel [45] ( ), El-Hawary [46] ( ), Hurly et al. [47] (◆), Evers et al. [48] (□), Abe et al. [54] ( ), Kestin et al. [55] ( ), Kestin et al. [56] ( ), Gough et al. [58] ( ), Kestin et al. [59] ( ), Timrot et al. [60] ( ), Hellemans et al. [64] (●), Maitland and Smith [61] ( ), Dawe et al. [65] (◯), Clarke and Smith [66] (◊)

One incentive for this work is incorporate *ab initio* results to improve the low-density behavior in regions where experimental data are unreliable or lacking, such as at the low (*T* < 150 K) and high (*T* > 700 K) temperature regions where high-accuracy experimental data are sparse. This was done by incorporating Eqs. 2–5 for densities below about 2 mol·L^−1^ (32 kg·m^−3^). Figure 2 demonstrates the behavior of the zero-density correlations, showing the increased deviations for the correlations of Quiñones-Cisneros et al. [7] and Vogel et al. [2] in regions where experimental data were lacking. We can also compare with experimental data. Although we have identified a primary dataset, the correlation was developed such that the viscosity is dominated by the dilute-gas correlation and the initial density dependence, Eqs. 2–5, for the region where the density is less than 2 mol L^−1^; the residual contribution is minimal in this region. Figure 5 shows comparisons with the most reliable low-uncertainty measurements at pressures up to 1 MPa for the present correlation, Eqs. 1–6, and for the Quiñones-Cisneros et al. [7] correlation. Vogel [45] published two series of measurements that cover the temperature range 292 K to 682 K; series 1 was an isochore at 0.344 kg m^−3^, while series 2 was taken at 0.498 kg m^−3^. These two series do not differ by more than 0.1 % except for two points in series 2 that Vogel notes were affected by electrical charging. The uncertainty given for Vogel’s measurements is 0.15 % at room temperature, rising to 0.2 % at the highest temperature. Series 1 however is systematically higher than series 2. The correlation of Quiñones-Cisneros et al. [7] reproduces series 2 (with the exception of the points affected by electrical charging) to within their stated uncertainty and exceeds the uncertainty for series 1 only slightly. Our correlation, however, has larger deviations than the Quiñones-Cisneros et al. [7] model due to our use of the *ab initio* based dilute-gas correlation of Xiao et al. [10]. Laesecke and Muzny [128] also observed this; pointing out that their correlation represented series 2 to within the uncertainty stated (except for the points with electrical charging, a possible outlier at 518.8 K, and the two highest temperature points), while series 1 is between 0.07 and 0.15 % higher than series 2. The re-evaluated data of May et al. [9] presented in Xiao et al. [10] are represented by both models to within their uncertainty, 0.134 % (at *k* = 2); these are presently the best data available and cover the temperature range 211 K to 392 K. The re-evaluated data of Schley et al. [42] given in Herrmann and Vogel [41] and the recent data of Humberg et al. [43] are also represented well by both models, even though Humberg et al. [43] was not available to Quiñones-Cisneros et al. [7]. As noted by Vogel [2], the data of Evers et al. [48] are higher than Vogel [2] for unknown reasons, as well as the higher temperature data of El-Hawary [46]. The dilute-gas correlation of Xiao et al. [10] has an average relative uncertainty of 0.2 % (at *k* = 2) over the range of the reference correlation, 80 K to 1500 K, ranging from 0.4 % at 100 K decreasing to a minimum of 0.0009 % at 290 K, and rising to 0.2 % at 900 K. The contribution from the initial density dependence term adds additional uncertainty to the correlation. Based on comparisons with experimental data and with the uncertainties given for the dilute-gas function, for the region *p* < 1 MPa, we estimate that our correlation, Eqs. 1–6, has an estimated uncertainty of 1 % from 90 K to 150 K, 0.3 % from 150 K to 210 K, 0.134 % for 210 K to 392 K, 0.2 % for 392 K to 700 K, 0.5 % for 700 K to 1100 K, and 1 % to 1500 K (the upper limit of the *ab initio* calculations). Additional accurate data in the high-temperature region (above 700 K) and in the low-temperature region (below 150 K) would allow improved assessments of the uncertainty in this region.

**Fig. 6:**
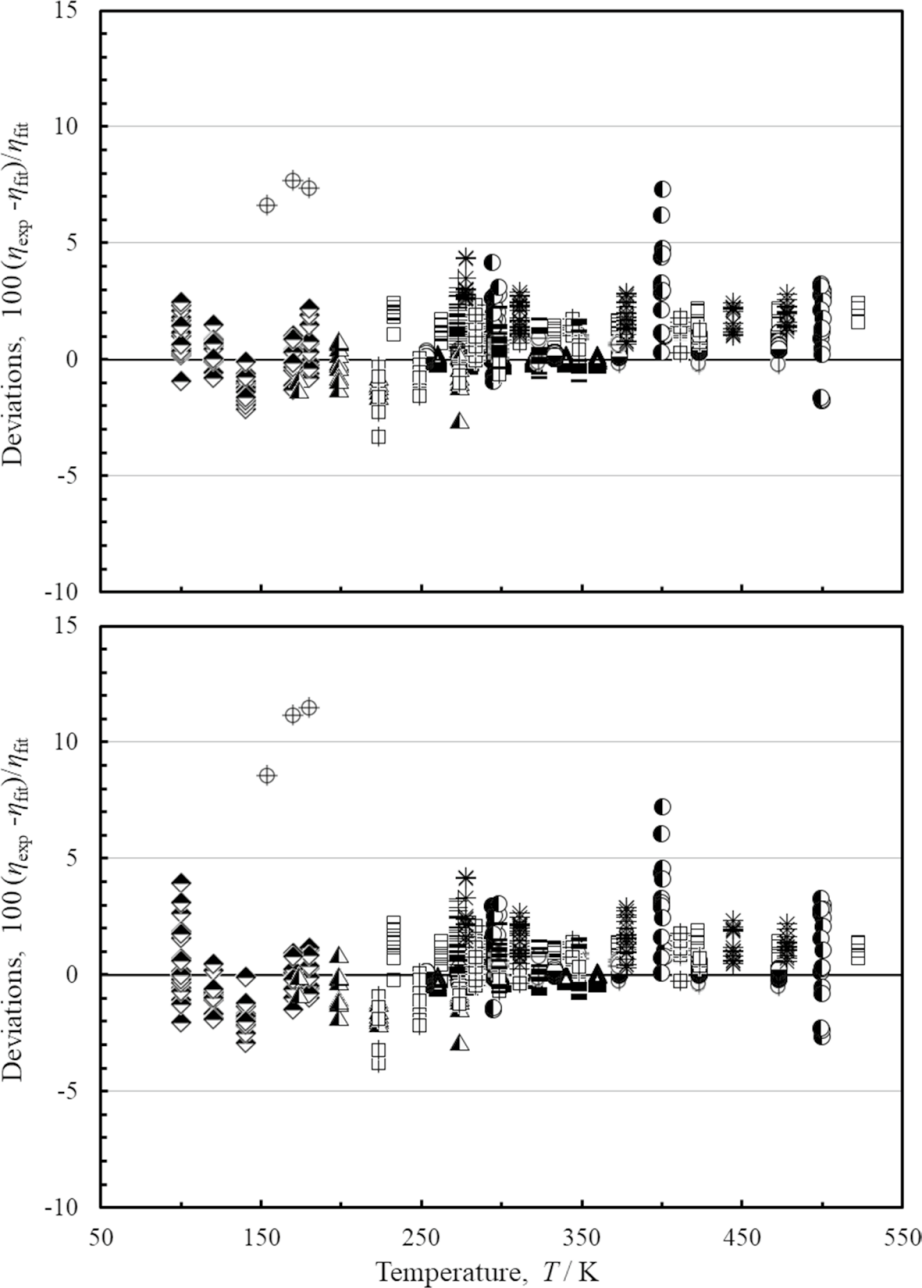
Percentage deviations of the primary data pressures between 1 and 100 MPa as a function of temperature calculated by the present model and the model of Quiñones-Cisneros et al. [7]. Top panel, this work, bottom panel, Quiñones-Cisneros et al. [7]. Herrmann and Vogel [41] (∆), Humberg et al. [43] ( ), El-Hawary [46] ( ), Hurly et al. [47] (◆), Evers et al. [48] (□), van der Gulik et al. [50] (+), Diller and Frederick [51] ( ), Hongo et al. [52] ( ), Diller [53] ( ), Chuang et al. [57] ( ), Timrot et al. [60] ( ), Slyusar et al. [62] ( ), Giddings et al. [68] ( ), Carmichael et al. [69] ( ), Barua et al. [70] ( ), Iwasaki et al. [71] (–)

Figure 6 shows deviations from the primary experimental data as a function of temperature for both models over the mid-pressure range of 1 MPa < *p* < 50 MPa. As indicated in Fig. 6 and in Table 6, both models are comparable in the mid-pressure range. In this pressure range the most accurate data are the re-evaluated data of Schley [42] given in Herrmann and Vogel [41]. These cover the temperature range 260 K to 360 K at pressures up to 29 MPa. The present model and the Quiñones-Cisneros et al. [7] model both represent this data to within the estimated experimental uncertainty, 0.3 %. For temperatures lower than 260 K there are two significant datasets, Chuang et al. [57] and Diller [53]. Comparisons with these data indicate that the present correlation has an estimated uncertainty of 2.5 % for pressure up to 50 MPa. For temperatures above 360 K, for pressures from 1 to 10 MPa the most accurate data are from Humberg et al. [43], El-Hawary [46] and Evers et al. [48]. Comparisons with these data indicate an uncertainty of 0.8 % for temperatures from 360 K to 523 K. For the pressure range of 10 to 30 MPa, comparisons with El-Hawary et al. [46] and Evers et al. [48] give an uncertainty of 1.5 %, again for temperature from 360 K to 523 K. For the higher pressures of 30 MPa to 50 MPa only the older data of Carmichael et al. [69], Giddings et al. [68], and Diller [53] are available for comparison for temperatures from 360 K to 500 K, and unfortunately these sets do not agree with each other to within their claimed uncertainties. Future measurements in this region could clarify behavior in this region. However, comparisons with these data for temperatures from 360 K to 500 K indicate an estimated uncertainty of 2 % in this region.

**Fig. 7:**
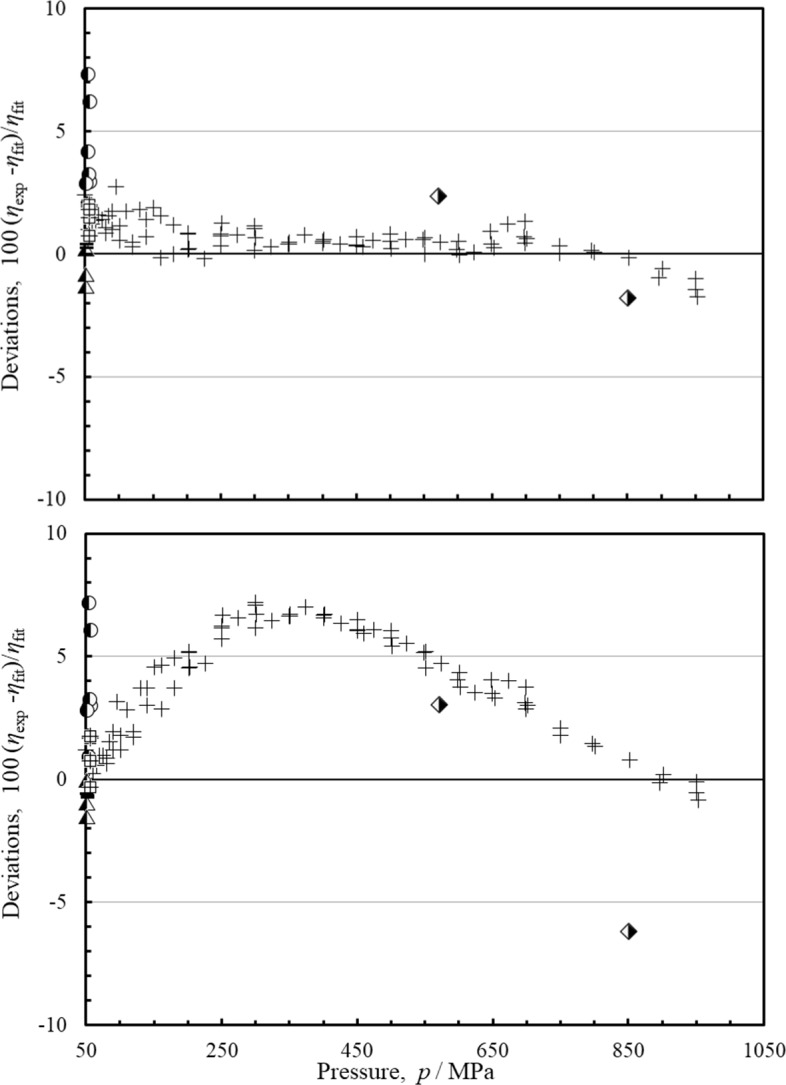
Percentage deviations of the primary data pressures between 50 and 1000 MPa as a function of pressure, calculated by the present model and the model of Quiñones-Cisneros et al. [7]. Top panel, this work, bottom panel, Quiñones-Cisneros et al. [7]. Abramson [44] ( ), van der Gulik et al. [50] (+), Diller and Frederick [51] ( ), Chuang et al. [57] ( ), Giddings et al. [68] ( ), Iwasaki et al. [71](–)

Figure 7 shows comparisons with data for the high-pressure region, from 50 MPa to 1000 MPa, the upper limit for pressure of the EOS. For the highest pressures, data for comparisons are limited. The measurements of van der Gulik et al. [50] extend to 953 MPa, but they are at a single temperature of 273 K. There are a few limited points around 50 MPa from Diller and Frederick [51], Chuang et al. [57], Giddings et al. [68], and Iwasaki and Takahashi [71] that overlap with van der Gulik et al. [78]. The Diller and Frederick data [51] have larger deviations than the other data in this region. Abramson [44] provides data over a wider temperature range (294 K to 673 K) and at pressures from 570 MPa to 6260 MPa; however, only two of the points are within the limits of the EOS. As mentioned earlier, the data of Abramson were used in the fitting process mainly to control extrapolation behavior. In addition, Abramson [44] did not provide an uncertainty estimate for his measurements. The current model offers some improvement for the high-pressure region, although additional high-accuracy, comprehensive measurements in this region would allow a better estimation of the uncertainty in this region and allow future improvements in a correlation. We estimate the uncertainty of the correlation in the pressure range from 50 MPa to 1000 MPa for temperatures from 223 K to 625 K is 4 %, with larger uncertainty at temperatures below 223 K.

**Fig. 8:**
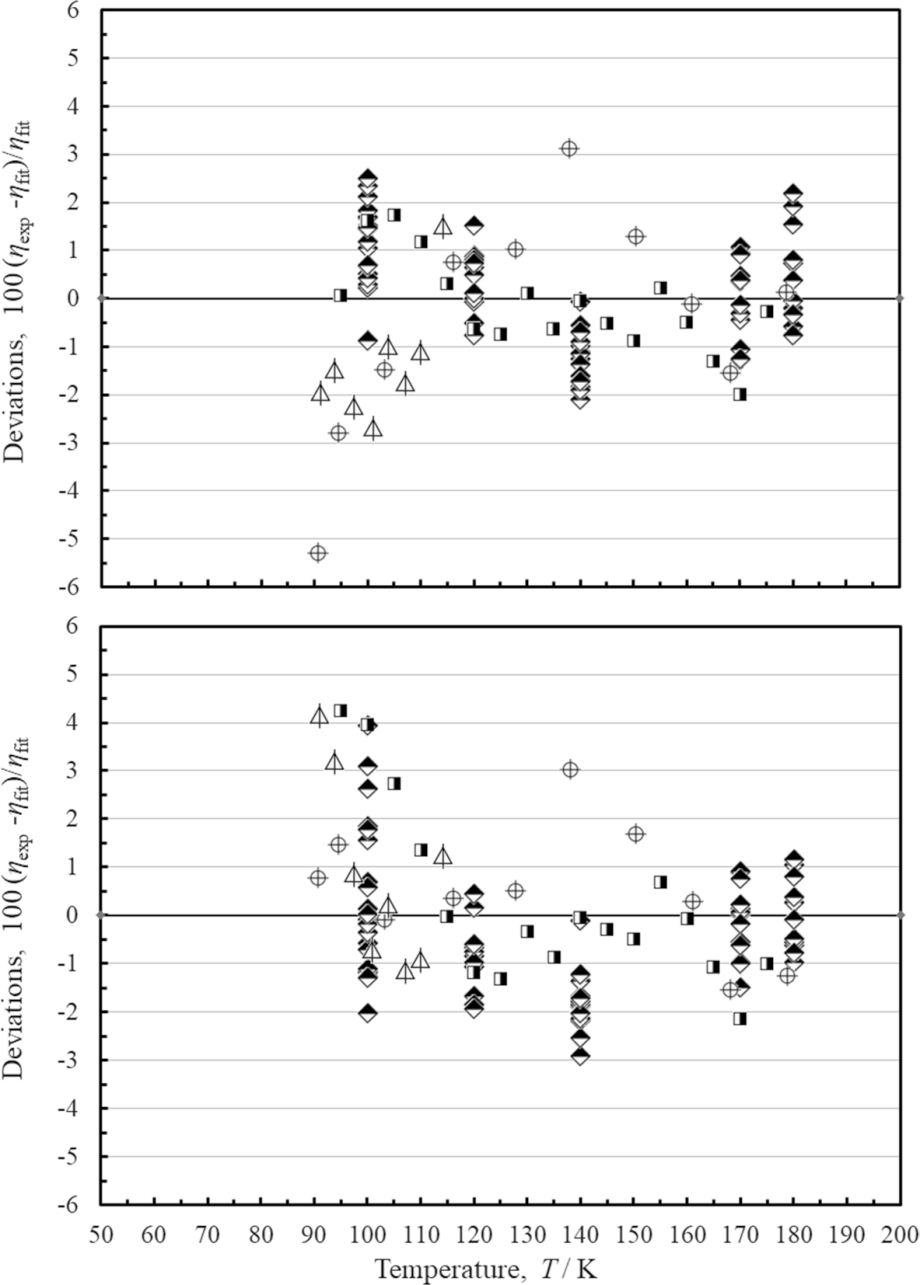
Percentage deviations of the primary data in the liquid phase at pressures up to 33 MPa calculated by the present model and the model of Quiñones-Cisneros et al. [7]. Top panel, this work, bottom panel, Quiñones-Cisneros et al.[7]. Diller [53] ( ), Slyusar et al. [62] ( ), Haynes [63] ( ), Boon et al. [67] ( )

Figure 8 shows deviations for both correlations for liquid-phase measurements. These extend up to 33 MPa. The performance of both correlations is similar. The estimated uncertainty for the liquid phase at pressures up to 33 MPa is 3 %.

**Fig. 9:**
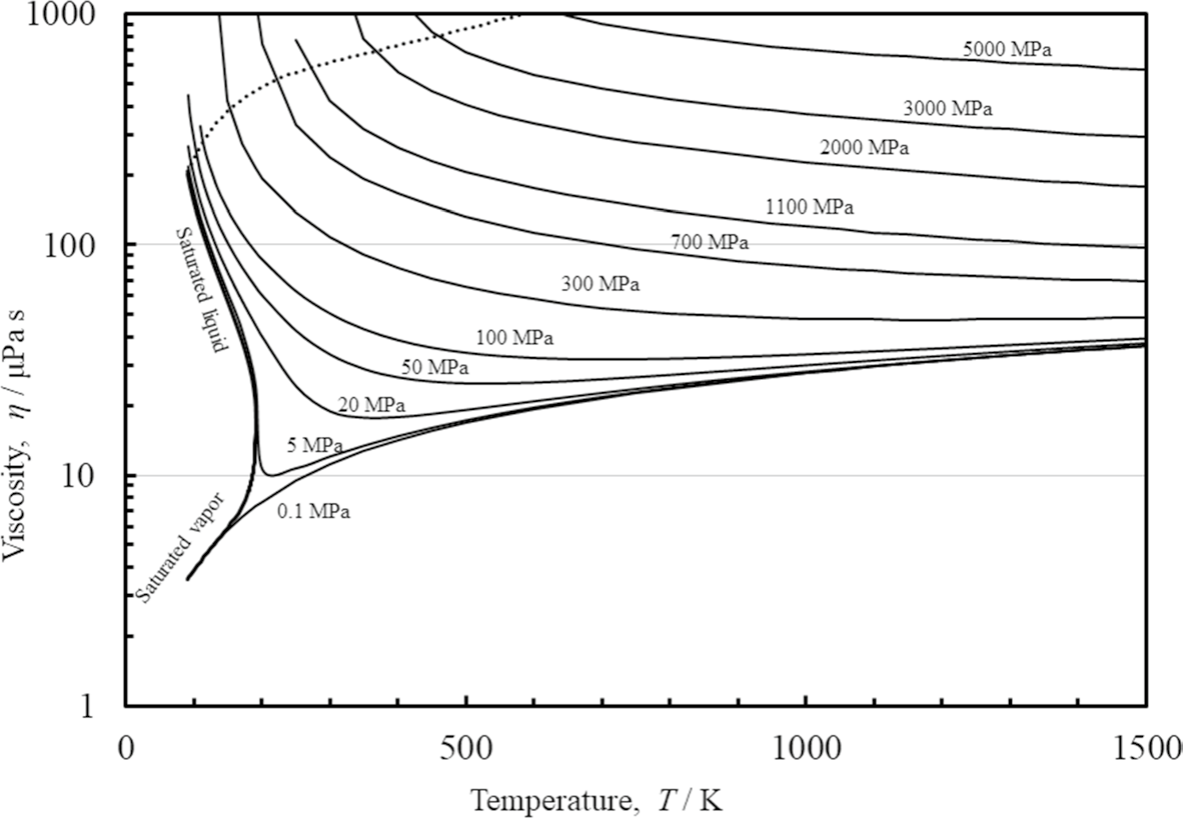
Viscosity of methane as a function of the temperature for different pressures. The extrapolated melting line by Setzmann and Wagner [32] is indicated by the dotted line

Finally, Fig. 9 shows a plot of the viscosity of methane as a function of the temperature for different pressures. The plot demonstrates the reasonable extrapolation behavior at pressures up to 5 GPa and temperatures to 1500 K, that well exceed the limits of the EOS by Setzmann and Wagner [32] (625 K and 1 GPa). The high-pressure data of Abramson [44] were used to ensure physically reasonable extrapolation behavior. For regions where there are no experimental data or theoretical guidance, we can only state that the behavior is physically reasonable (no discontinuities, and the isobars on the temperature-viscosity plot do not cross). The extrapolated melting line of Setzmann and Wagner [32] is indicated by the dotted line, and the correlation does not have unphysical behavior such as mathematical poles within the fluid region. As noted earlier, the correlation does exhibit unphysical behavior outside of this region and users should be aware of this possibility.

## Recommended Values and Computer-Program Verification

### Recommended Values

In Table 8, viscosity values are given along the saturation boundary, calculated from the present proposed correlation between 100 K and 190 K, while in Table 9, viscosity values are given for temperatures between 100 K and 600 K and at selected pressures. Saturation density values for selected temperatures, as well as the density values for the selected temperature and pressure are obtained from the equation of state of Setzmann and Wagner [32]. The values in the tables are calculated from the given temperatures and densities according to Eqs. 1–6.

**Table 8:** Viscosity values of methane along the saturation boundary, calculated by the present scheme

*Τ* (Κ)	*ρ*_liq_ (kg·m^−3^)	*ρ*_vap_ (kg·m^−3^)	*η*_liq_ (μPa·s)	*η*_vap_ (μPa·s)
100	438.89	0.67457	154.50	3.857
110	424.78	1.5982	121.15	4.227
120	409.90	3.2619	98.180	4.608
130	394.04	5.9804	80.976	5.005
140	376.87	10.152	67.387	5.433
150	357.90	16.328	56.233	5.913
160	336.31	25.382	46.763	6.487
170	310.50	38.974	38.392	7.244
180	276.23	61.375	30.406	8.447
190	200.78	125.18	19.320	12.451

**Table 9:** Viscosity values of methane at selected temperatures and pressures, calculated by the present scheme

*p* (MPa)	*T* (K)	*ρ* (kg·m^−3^)	*η* (μPa·s)
0.1	100	438.94	154.6185
	150	1.306	5.7875
	200	0.971	7.6787
	400	0.483	14.1711
	600	0.322	19.3488
10	100	446.02	173.5911
	150	375.63	66.1589
	200	266.19	29.2215
	400	49.736	15.6357
	600	31.474	20.0752
50	100	468.27	273.4839
	150	417.69	100.8833
	200	366.92	60.2015
	400	203.43	26.5069
	600	134.47	25.1522
100	100	488.65	589.2895
	150	447.37	141.6910
	200	409.03	86.7720
	400	286.86	38.5076
	600	214.28	32.2006
200	100	Solid	Solid
	150	485.31	241.8805
	200	456.01	137.7745
	400	363.87	59.4917
	600	300.89	45.1688
500	100	Solid	Solid
	150	Solid	Solid
	200	529.46	356.9237
	400	464.11	121.8527
	600	416.06	84.8895
1000	100	Solid	Solid
	150	Solid	Solid
	200	Solid	Solid
	400	545.35	239.0720
	600	506.59	159.7075

### Computer-Program Verification

For checking computer implementations of the correlation, the following points may be used for the given *T*, *ρ* conditions: *T* = 300 K, *ρ* = 0 kg·m^−3^, *η* = 11.1230 μPa·s, *T* = 300 K, *ρ* = 3.2 kg·m^−3^, *η* = 11.1891 μPa·s, and *T* = 300 K, *ρ* = 75 kg·m^−3^, *η* = 13.7130 μPa·s. In addition, a small Python program is included in the Supporting Information to reproduce these values.

## Conclusions

A new, wide-ranging correlation for the viscosity of methane based on critically evaluated experimental data was presented. This correlation is designed to be used over the range of applicability of the equation of state of Setzmann and Wagner [32] that extends from the triple-point temperature (90.6941 K) to 625 K, at pressures up to 1000 MPa. It uses the dilute-gas correlation of Xiao et al. [10] that incorporates *ab initio* calculations, and an initial density dependence based on Rainwater–Friend [33, 34] theory. The residual contribution is empirical and was obtained using open-source symbolic regression software TiSR developed by Martinek and coworkers [135, 136]. The estimated uncertainty of the correlation (at *k* = 2) varies from a low of 0.134 % for the gas at pressures below 1 MPa over temperatures from 210 K to 392 K, to 0.8 % to 2 % depending on the temperature for the mid-pressure range of 1 MPa < *p* < 50 MPa, and is 4 % for pressures from 50 MPa to 1000 MPa for temperatures from 223 K to 625 K. In the liquid region at pressures up to 33 MPa, the estimated uncertainty is 3 %.

## Supplementary information

Supplementary file METHANE.FLD file contains the parameters for the calculation of the thermophysical properties of methane including the viscosity correlation in this work for use with the REFPROP v10.0 [11] computer program and is provided. This file also is available online at 10.18434/mds2-3972.

In addition, a small Python program is included that reproduces the computer checking values.

## Supplementary Information

Below is the link to the electronic supplementary material.

Supplementary file1 (ZIP 14 kb)

## Data Availability

Supplementary file METHANE.FLD file contains the parameters for the calculation of the thermophysical properties of methane including the viscosity correlation in this work for use with the REFPROP v10.0 [ [11](.)] computer program and is provided. This file also is available online at 10.18434/mds2-3972. In addition, a small Python program is included that reproduces the computer checking values.
